# Survey datasets on the influence of conflict management strategies on academic staff productivity in selected public universities in Nigeria

**DOI:** 10.1016/j.dib.2018.04.139

**Published:** 2018-05-05

**Authors:** Ebeguki Igbinoba, Hezekiah Falola, Adewale Osibanjo, Olumuyiwa Oludayo

**Affiliations:** Business Management, Covenant University, Nigeria

**Keywords:** Conflict, Conflict management strategies, Productivity, Public universities, Nigeria

## Abstract

The article presented data on the influence of conflict management strategies on productivity of Academic Staff of selected public universities in Nigeria. A total of 368 copies of questionnaire were administered to the academic staff of selected three (3) Public Universities in the South–West Zone of Nigeria and 325 copies were returned and deemed usable. This represents a response rate of 88.32%. Emphasis was laid on the year of establishment of the selected universities based on the fact that over the years, these universities are likely to have been exposed to conflicts and the strategies for managing them. The study adopted the quantitative approach with a descriptive research design to establish trends related to the objectives of the study. The questionnaire used for this study was adapted from the work of Rahim (2002) [4]. The population of this study included all the Academic Staff of the selected public universities. Data was analyzed with the use of multiple regression and structural equation modeling. The data set is made available and accessible for more comprehensive research.

## Specification Table

**Table:** 

**Subject area**	Labour Relations, Conflict Management
**More Specific Subject Area:**	Industrial Relations and Human Resource Management
**Type of Data**	Table
**How Data was Acquired**	Data were collected through questionnaire
**Data format**	Raw, analyzed, Inferential statistical data
**Experimental Factors**	Sample consisted of 368 Academic Staff of some selected public universities.
**Experimental features**	Sample selection of the views of the Academic Staff of selected Public Universities on the strategic management of conflicts and how they affect their levels of productivity
**Data source location**	Nigeria
**Data Accessibility**	Data is included in this article

## Value of data

● Primarily, the data is about perennial issues of conflicts in Nigerian Public Universities, which often result in wasted academic sessions and even loss of property and lives, thus making it a very serious national problem.
● The data presented will enable universities management to have proper understanding and better insights into what causes conflicts and how they can be effectively managed.
● The understanding of government agencies and other stakeholders in the education sector will be enhanced and thereby, empowered to formulate policies that will reduce the rampant cases of conflicts.
● The data provides insights into diverse aspects of conflict management in the university system.
● Academics will be provided with a platform upon which to advance further research on the related subject matters.
● Confidence in the generalisation of the results of further analysis is enhanced by the spread of the universities and the sample of academic staff involved in the study.

## 1 Data

**Fig. 1:**
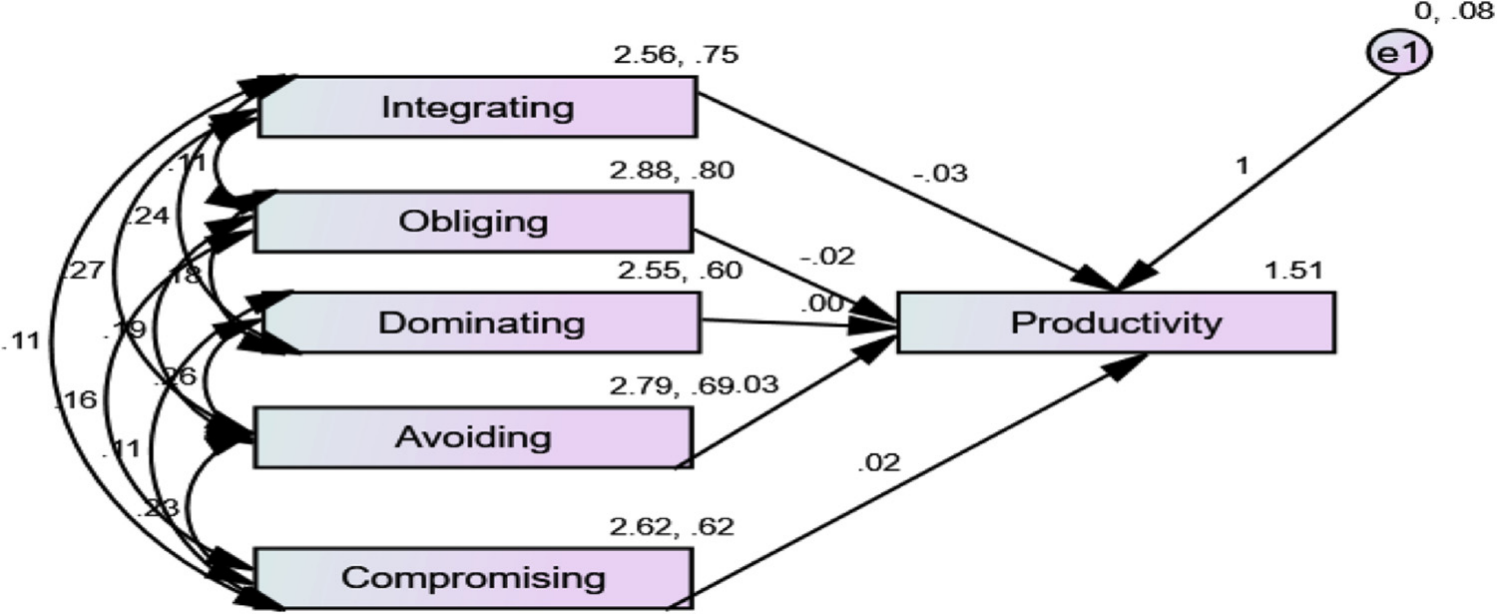
Conflict management strategies and academic staff productivity model.

The study provided primary data on the influence of conflict management strategies on academic staff productivity in some selected public universities in Nigeria. Multiple regression analysis was used to determine the predictive capability of the various conflict resolution strategies. Table 1 shows the coefficient analysis of conflict management strategies on staff productivity while Fig. 1 shows the output of structural equation modeling of the processed data. The coefficient table and structural equation model provide information about the predictive capabilities as depicted in Table 1 and Fig. 1.

**Table 1:** Coefficients of conflict management strategies on staff productivity.

Coefficients^a^						
Model		Unstandardized coefficients		Standardized coefficients	*T*	Sig.
		*B*	Std. Error	Beta		
1	(Constant)	2.323	.108		21.464	.000
	Integrating	− .102	.030	− .223	− 3.407	.001
	Obliging	−.070	.034	− .133	− 2.052	.041
	Dominating	.102	.034	.191	2.995	.003
	Avoiding	− .039	.031	− .083	− 1.257	.210
	Compromising	.011	.030	.024	.362	.718

a Dependent Variable: Productivity.

## 2 Experimental design, materials and methods

In this data presentation, a descriptive survey design was adopted. The sample for this study consisted of 368 respondents who were randomly drawn from (3) selected Public Universities in Nigeria. Data were gathered from the sample of the Academic Staff of the selected universities across the various faculties with the aid of an adapted questionnaire developed by [1], [3], [4]. Data collected were collated, coded and inputted into SPSS-22 and AMOS-22 was used for the analysis of the multiple regression and structural equation modelling The study provides useful insights into what the management of the institutions of higher learning can do to manage conflicts strategically, thereby improving the harmonious relationship between management and Academic Staff of public universities. Details on the conflict management strategies and academic staff productivity can be accessed in [2].

## 3 Conclusion and implications for the study

This study revealed that conflict management strategies have significant and positive impact on academic staff productivity of the selected public universities in Nigeria. The study revealed that selected public Universities use a dominating strategy for influencing academic staff productivity. The success of these universities depend on the ability to ensure that the decision they make is in line with the goals, values and philosophies of the institutions. Hence, this study has extensive implications for institutions, academic staff, government, educators and researchers in this regard. To this end, the data presented in this article is imperative for more comprehensive analysis or investigation.

## 4 Ethical considerations

The researchers ensured that respondents were well informed about the study and the objectives of this research and they were encouraged with the participation process. Respondents were offered the opportunity to stay anonymous and their responses were treated confidentially.
