# Supplementary material for: Survey datasets on the influence of conflict management strategies on academic staff productivity in selected public universities in Nigeria
Source: Data Brief. 2018 May 5;19:322–5. doi: 10.1016/j.dib.2018.04.139 (PMC5992974; doi:10.1016/j.dib.2018.04.139)
Supplement: Supplementary file 1 — Supplementary material [file mmc1.docx]

**DECLARATION OF INTEREST FORM**

**Survey Datasets on the Influence of Conflict Management Strategies on Academic Staff Productivity in some Selected Public Universities in Nigeria**

Ebeguki **IGBINOBA**; Covenant University

ebe.igbinoba@covenantuniversity.edu.ng

Hezekiah **FALOLA**; Covenant University

[hezekiah.falola@covenantuniversity.edu.ng](mailto:hezekiah.falola@covenantuniversity.edu.ng)

Adewale **OSIBANJO**; Covenant University

adewale.osibanjo@covenantuniversity.edu.ng

Olumuyiwa **OLUDAYO**; Covenant University

[olumuyiwa.oludayo@covenantuniversity.edu.ng](mailto:olumuyiwa.oludayo@covenantuniversity.edu.ng)

We, the Authors of paper entitled above certify that we have seen and approved the final version of the manuscript being submitted. This is an original work and has not received prior publication and is not under consideration for publication elsewhere. It is also important to state that there is no financial/personal interest or belief that could affect our objectivity and to prevent ambiguity, we humbly want to state explicitly that there is no conflicts of interest as regards the review and publication of this paper.

Thank you.

IGBINOBA Ebeguki Edith

*Signed*
